# Association of Radioiodine for Differentiated Thyroid Cancer and Second Breast Cancer in Female Adolescent and Young Adult

**DOI:** 10.3389/fendo.2021.805194

**Published:** 2022-01-28

**Authors:** Xianlan Zhao, Mingjing Chen, Xiaojing Qi, Haizhen Zhu, Guangrong Yang, Yi Guo, Qiang Dong, Qiao Yang

**Affiliations:** ^1^ People's Hospital of Honghuagang District, Zunyi, China; ^2^ Department of Infectious Diseases, The First Affiliated Hospital of Chongqing Medical University, Chongqing, China; ^3^ Department of Ultrasound, The 941st Hospital of the People’s Liberation Army (PLA) Joint Logistic Support Force, Xining, China; ^4^ Department of Oncology, Guizhou Provincial People’s Hospital, Guizhou Cancer Center, Guiyang, China; ^5^ Department of Oncology, Qijiang Hospital of the First Affiliated Hospital of Chongqing Medical University, Qijiang, China; ^6^ Department of Basic Knowledge, Guiyang Nursing Vocational College, Guiyang, China; ^7^ Department of General Medicine, Qijiang Hospital of the First Affiliated Hospital of Chongqing Medical University, Qijiang, China

**Keywords:** differentiated thyroid cancer, second breast cancer, adolescent and young adult, radioiodine, SEER

## Abstract

**Background:**

Exposure to radiation is related to breast cancer occurrence. While whether the radioiodine (RAI) increases the risk of second breast cancer (SBC) in female differentiated thyroid cancer (DTC) patients is not well addressed.

**Methods:**

All patients were identified from Surveillance, Epidemiology, and End Results database. At least a 5-year latency was guaranteed since exposure to RAI. Fine and Gray model was used to calculate the cumulative incidence and hazards ratios (HR) and 95% confidence interval (CI). Standardized incidence ratio (SIR) was calculated by Poisson regression analysis. Propensity score matching was used for match analysis. Survival analyses were performed by the Kaplan-Meier method and the log-rank test.

**Results:**

A total of 406 out of 16,850 patients in the RAI group and 733 out of 22,135 patients in the no RAI group developed SBC. The cumulative incidences of SBC were higher in patients with RAI compared with patients without RAI in the adolescent and young adult (AYA) group and the middle-aged adult group. In the AYA group, patients with RAI had increased HR (1.65; 95% CI, 1.33–2.05, *p* < 0.001) compared with those without RAI, and the HR increased slightly with latency. In addition, the SIR (1.21; 95% CI, 1.02–1.44, *p* < 0.05) increased compared with the general population. Whereas, in the middle-aged adult group, only a slightly higher HR (1.18) was found. The survival after SBC was inferior to those with matched only primary breast cancer.

**Conclusions:**

RAI treatment increased the risk of SBC in female AYA DTC patients. A long-term follow-up should be performed in this population.

## Introduction

The incidence of thyroid cancer increased sharply over the past decades (1), and the differentiated thyroid cancer (DTC) accounted for more than 90% of all thyroid cancer (2). With the treatment of surgery and radioiodine [RAI, i.e., iodine-131 (I-131)] therapy, a 10-year overall survival (OS) exceeding 90% was found in DTC patients (3). The excellent long-term survival and increased incidence of DTC raised the risk of second primary malignancy (SPM) as rare but notable intermediate and late effects.

It has been reported that an increased risk of developing solid SPM was related to RAI treatment in DTC patients (4, 5). This raises the concern of second breast cancer (SBC) after RAI treatment of female DTC patients, because of the mammary gland could uptake the iodide, and the exposure to radiation could raise the risk of developing breast cancer (6). A careful assessment of the benefits and risks of RAI treatment for female DTC patients should be performed during the medical decision process (7).

According to a review (8), a few studies have investigated the risk of SBC in DTC patients with RAI treatment, while the results are contradictory. Most studies had limited samples or not enough follow-up time. Moreover, the risks among different age groups are not well addressed. Previous studies referred mainly to the middle-aged and older adult patients, few studies focused on the adolescent and young adult (AYA) patients. The aim of this study was to determinate the risk of SBC after RAI in female DTC patients, especially in the AYA patients, by a large population from the Surveillance, Epidemiology, and End Results (SEER) database. In addition, we assessed the survival outcomes after SBC.

## Methods

### Database, Participants, and Variables

From January 1, 1975 to December 31, 2011, female patients with DTC as the first primary malignancy (FPM) were identified from SEER 9 registries. The selecting criterion included the following: tumor located in thyroid gland, female gender, age ≥15 years, microscopically confirmed, and type of reporting source is not autopsy only or death certificate only. The histology codes were coded according to the *International Classification of Diseases for Oncology, Third Edition (ICD-O-3)*. The papillary cancer included 8,050/3, 8,052/3, 8,130/3, 8,260/3, 8,340–8,344/3, 8,450/3, and 8,452/3, and the follicular cancer included 8,290/3, 8,330–8,332/3, and 8,335/3. The details of subgroups of each variable were described as follows: age (15–39 years/AYA group, 40–69 years/middle-aged adult group, ≥70 years/older adult group), race (white, black, others), histology (follicular, papillary), tumor grade (I/II, III/IV, unknown), and SEER stage (localized, regional, distant, unknown).

Patients with/or combined with beam radiation treatment were not included in this study. Then all identified patients were classified into two groups according to initial RAI treatment, with RAI and without RAI. The study design is presented in **Supplementary Figure S1**.

### Outcome Measurement

The first primary outcome was the cumulative incidence of SBC in female DTC survivors, with at least 60 months of latency. Because at least a 5-year latency from radiotherapy exposure to solid tumor occurrence was considered to be radiation-induced cancer (9). Latency was defined as the time interval between diagnosis of DTC and diagnosis of SBC. The SEER database uses a set of multiple primary rules to distinguish SPM from recurrence. The second outcome was the OS, which was defined as the follow-up time from diagnosis of SBC to death due to any reason in female DTC patients or the follow-up time from diagnosis to death due to any reason in the matched only primary breast cancer (PBC) patients. The last follow-up time was December 31, 2016; patients who were alive at the last follow-up were regarded as censored cases.

### Statistical Analysis

Considering any reason of death and developing other SPMs as competing events, Fine and Gray competing risk regression was used to calculate the cumulative incidence of developing SBC, as well as the hazard ratio (HR) and 95% confidence interval (CI) for SBC occurrence in the univariable and multivariable analyses. To better evaluate the risk for developing SBC, standardized incidence ratio (SIR) was calculated by Poisson regression analysis. The SIR was defined as the ratio of observed incidence of SBC in DTC patients to the incidence of SBC in US general population. In addition, the HRs and SIRs were stratified by the latency to show the dynamic changes.

Survival analysis was performed by the Kaplan-Meier method and the log-rank test. Propensity score matching (PSM) was used to match each SBC patient after RAI/no RAI with only five PBC patients for further survival analysis. The following predetermined variables were considered for matching, including age at breast cancer diagnosis, race, tumor grade, and SEER stage.

Pearson’s chi-square test was used to compare categorical data, and Mann-Whitney *U* test was used to compare continuous data. All cases were identified with SEER*Stat software (version 8.3.9; https://seer.cancer.gov/seerstat/). All statistical analyses were performed with R software (version 4.0.5; http://www.r-project.org/). The SIRs were calculated with SEER*Stat software. Two-sided p-value <0.05 was considered to indicate statistically significant difference.

## Results

### Clinical Features of Patients

A total of 38,985 female DTC patients were identified from SEER 9 registries between 1975 and 2011. The AYA group accounted for 41%, and 53% for the middle-aged adult group and 6% for the older adult group. Among them, 31,694 (81%) were white and 34,908 (90%) were papillary cancer. The proportions of localized and regional were 65% and 31%, while distant only accounted for 2%. The median follow-up time was 162 months, with an interquartile range (IQR) of 103–259 months. All patients were classified into two groups according to receiving RAI treatment or not. After at least a latency of 60 months, 406 out of 16,850 (2.4%) patients in the RAI group and 733 out of 22,135 (3.3%) patients in the no RAI group developed SBC. The details of baseline clinical features and comparison between patients with and without RAI are shown in **Table 1**.

**Table 1:** Baseline clinical feature comparison between differentiated thyroid cancer patients with and without radioiodine.

Variables	Total [*N* = 38,985 (%)]	DTC patients without RAI [*N* = 22,135 (%)]	DTC patients with RAI [*N* = 16,850 (%)]	*p*-value
**Age (years)**				
15–39	1,6171 (41)	8,906 (40)	7,265 (43)	<0.001
40–69	20,597 (53)	11,804 (53)	8,793 (52)	
≥70	2,217 (6)	1,425 (6)	792 (5)	
**Race**				
White	31,694 (81)	18,290 (83)	13,404 (80)	<0.001
Black	2,356 (6)	1,435 (6)	921 (5)	
Others	4,935 (13)	2,410 (11)	2,525 (15)	
**Histology**				
Papillary	34,908 (90)	19,810 (89)	15,098 (90)	0.747
Follicular	4,077 (10)	2,325 (11)	1,752 (10)	
**Tumor grade**				
I/II	6,812 (17)	3,792 (17)	3,020 (18)	0.002
III/IV	258 (1)	124 (1)	134 (1)	
Unknown	31,915 (82)	18,219 (82)	13,696 (81)	
**SEER stage**				
Localized	25,515 (65)	16,662 (75)	8,853 (53)	<0.001
Regional	12,017 (31)	4,673 (21)	7,344 (44)	
Distant	759 (2)	243 (1)	516 (3)	
Unknown	694 (2)	557 (3)	137 (1)	
**Follow-up time (median (IQR), months)**	162 (103–259)	185 (119–278)	158 (101–255)	<0.001

DTC, differentiated thyroid cancer; RAI, radioiodine; SEER, Surveillance, Epidemiology, and End Results; IQR, interquartile range.

### Cumulative Incidences and Risk Factors of Developing SBC

**Figure 1:**
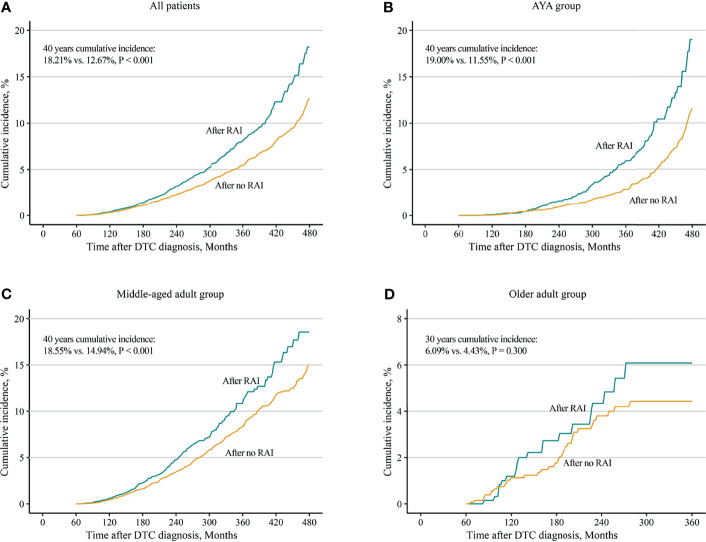
Comparisons of cumulative incidences of second breast cancer in female differentiated thyroid cancer patients with radioiodine and with no radioiodine. **(A)** All patients, **(B)** AYA group, **(C)** middle-aged adult group, and **(D)** older adult group. RAI, radioiodine; DTC, differentiated thyroid cancer; AYA, adolescent and young adult.

Considering death and non-SBC as competing events, the overall 40 years cumulative incidence was 18.21% and 12.67% (*p* < 0.001) in patients with and without RAI, respectively (**Figure 1A**). Univariable and multivariable Fine and Gray competing risk regression analyses found that the adjusted HR for developing SBC was 1.27 [after RAI vs. after no RAI (95% CI, 1.13–1.44), *p* < 0.001; **Table 2**; **Supplementary Table S1**].

**Table 2:** Risk of developing second breast cancer in female-differentiated thyroid cancer patients.

Age group (years)	Multivariable competing risk regression (after RAI vs. after no RAI)		Poisson regression (after RAI vs. US general population)		Poisson regression (after no RAI vs. US general population)	
	Adjusted HR (95% CI)	*p*-value	SIR (95% CI)	*p*-value	SIR (95% CI)	*p*-value
All	1.27 (1.13–1.44)	<0.001	1.14 (1.03–1.25)	<0.05	1.03 (0.95–1.10)	>0.05
15–39	1.65 (1.33–2.05)	<0.001	1.21 (1.02–1.44)	<0.05	0.99 (0.87–1.12)	>0.05
40–69	1.18 (1.01–1.38)	0.032	1.09 (0.96–1.23)	>0.05	1.05 (0.96–1.15)	>0.05
≥70	1.24 (0.72–2.13)	0.440	1.25 (0.78–1.92)	>0.05	0.97 (0.67–1.36)	>0.05

RAI, radioiodine; HR, hazard ratio; CI, confidence interval; SIR, standardized incidence ratio.

Furthermore, analyses in different age groups were performed. The results turned out that both in the AYA group [19.00% vs. 11.55%, *p* < 0.001; HR, 1.65 (95% CI, 1.33–2.05), *p* < 0.001] and in the middle-aged adult group [18.55% vs. 14.94%; *p* < 0.001; HR 1.18 (95% CI, 1.01–1.38), *p* = 0.032], increased 40-year cumulative incidences (**Figures 1B, C**) and HRs (**Table 2**; **Supplementary Tables S2**, **S3**) were observed in patients with RAI. For the older adult group, because of a short life expectancy, only 30-year cumulative incidence was observed, and no differences of cumulative incidence (6.09% vs. 4.43%, *p* = 0.300; **Figure 1D**) and HR (1.24; 95% CI, 0.72–2.13, *p* = 0.440; **Table 2**; **Supplementary Table S4**) were found between patients with and without RAI. Furthermore, in subgroup analyses, the increased subdistribution hazards ratios (SHRs) of developing SBC were associated with RAI treatment in most subgroups of the AYA group (**Supplementary Figure S2**). While in the middle-aged adult group, increased SHRs were only found in subgroups of white, papillary, and regional (**Supplementary Figure S3**). No significant SHRs were found in the subgroups of the older adult group (**Supplementary Figure S4**).

When compared with US general population in analysis of developing SBC, only the AYA group with RAI treatment had an increased risk [SIR, 1.21 (95% CI, 1.02–1.44), *p* < 0.05]. The other two groups with RAI and all three groups without RAI had no significant SIRs. The details of SIRs are shown in **Table 2**.

### Dynamic Risk by Latency

**Figure 2:**
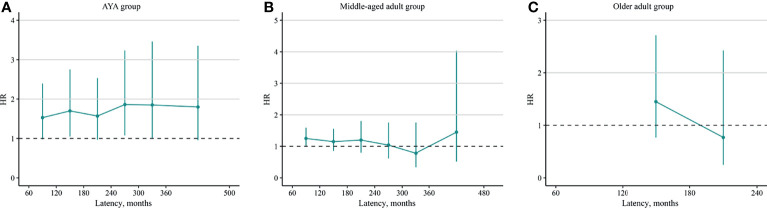
Dynamic radioiodine-related hazard ratio stratified by latency. **(A)** AYA group, **(B)** middle-aged adult group, and **(C)** older adult group. AYA, adolescent and young adult.

To further identify the dynamic changes of the risk, HRs and SIRs stratified by latency were calculated. In the AYA group, the HRs increased slightly with the increased in latency [60–119 months: HR, 1.53 [(95% CI, 0.98–2.39), *p* = 0.060; 120–179 months: HR, 1.70 (95% CI, 1.05–2.75), *p* = 0.031; 180–239 months: HR, 1.57 (95% CI, 0.97–2.53), *p* = 0.066; 240–299 months: HR, 1.86 (95% CI, 1.08–3.23), *p* = 0.026; 300–360 months: HR, 1.85 (95% CI, 0.99–3.46), *p* = 0.053; ≥360 months: HR, 1.80 (95% CI, 0.96–3.35), *p* = 0.066; **Figure 2A**]. In the middle-aged adult group, the HRs showed a decreasing tendency but no statistical significance [60–119 months: HR, 1.25 (95% CI, 0.99–1.59), *p* = 0.061; 120–179 months: HR, 1.15 (95% CI, 0.86–1.55), *p* = 0.350; 180–239 months: HR, 1.20 (95% CI, 0.80–1.80), *p* = 0.380; 240–299 months: HR, 1.04 (95% CI, 0.62–1.75), *p* = 0.880; 300–360 months: HR, 0.78 (95% CI, 034–1.75), *p* = 0.540; ≥360 months: HR, 1.45 (95% CI, 0.52–4.03), *p* = 0.470; **Figure 2B**]. In the older adult group, as few SBC cases were identified in latency of 60–120 and ≥240 months, the HRs during these periods could not be estimated. The HRs in latency of 120–179 months [1.45 (95% CI, 0.77–2.71), *p* = 0.250] and 180–239 months [0.77 (95% CI, 0.25–2.42), *p* = 0.660] showed no significant difference (**Figure 2C**).

Compared with US general population, the dynamic SIRs of SBC after RAI and SBC after no RAI showed no significant difference (**Supplementary Tables S5, S6**).

### Latency and Survival

**Figure 3:**
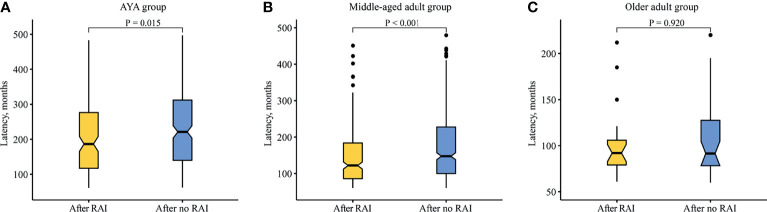
Comparison of latency between SBC patients after radioiodine and after no radioiodine. **(A)** AYA group. **(B)** Middle-aged adult group. **(C)** Older adult group. RAI, radioiodine; AYA, adolescent and young adult.

The latency difference between SBC after RAI and after no RAI in each age group was performed. The median latency of SBC after RAI was shorted than that of SBC after no RAI in the AYA group (186.5 vs. 221.0 months, *p* = 0.015; **Figure 3A**), as well as in the middle-aged adult group (122.0 vs. 147.5 months, *p* < 0.001; **Figure 3B**). While in the older adult group, the latency showed no difference (92.0 vs. 91.5 months, *p* = 0.924; **Figure 3C**).

**Figure 4:**
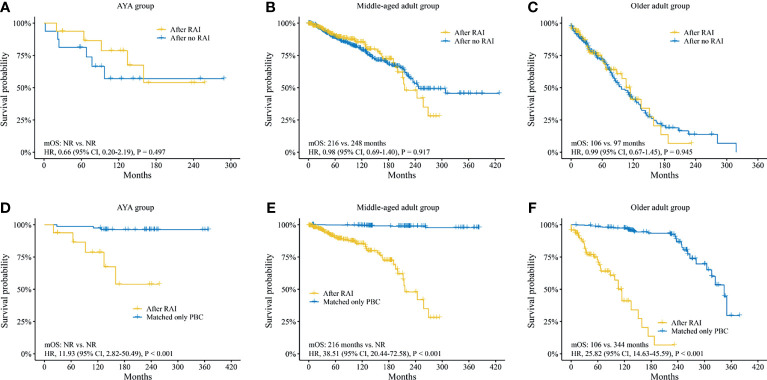
Survival analysis of second breast cancer. Survival analyses between SBC patients after radioiodine and after no radioiodine in **(A)** AYA group, **(B)** middle-aged adult group, **(C)** older adult group. Survival analyses between SBC patients after radioiodine and patients with matched only primary breast cancer in **(D)** AYA group, **(E)** middle-aged adult group, and **(F)** older adult group. SBC, second breast cancer; AYA, adolescent and young adult; RAI, radioiodine; OS, overall survival; NR, not reached; HR, hazard ratio; CI, confidence interval; PBC, primary breast cancer.

The median OS (mOS) of SBC patients between RAI group and no RAI group showed no differences in the AYA group [mOS not reached (NR) vs. NR; HR, 0.66 (95% CI, 0.20–2.19), *p* = 0.497; **Figure 4A**], the middle-aged adult group [mOS, 216 vs. 248 months; HR, 0.98 (95% CI, 0.69–1.40), *p* = 0.917; **Figure 4B**] and the older adult group [mOS, 106 vs. 97 months; HR, 0.99 (95% CI, 0.67–1.45), *p* = 0.945; **Figure 4C**].

In addition, the survival analyses between SBC patients after RAI and only PBC patients, as well as SBC patients after no RAI and only PBC patients were performed. In order to exclude the effect of clinical feature biases on the survival analysis, a 1:5 (SBC : PBC) PSM analysis was performed. A total of 406 SBC patients after RAI and 2,030 matched only PBC patients were confirmed. The clinical features showed no difference (**Supplementary Table S7**). Survival analysis found that the mOS of SBC patients after RAI were much inferior to that of matched only PBC patients in all three groups (**Figures 4D–F**). A total of 733 SBC patients after no RAI and 3,665 matched only PBC patients were confirmed. Neither of the clinical features showed any difference (**Supplementary Table S8**). The mOS of SBC patients after no RAI were also much poorer than that of matched only PBC patients (**Supplementary Figure S5A–C**).

## Discussion

In this population-based study, a comprehensive analysis regarding the risk and survival of SBC after RAI in female DTC patients was performed. Several key findings should be noted. First of all, the calculated cumulative incidences of AYA group and middle-aged adult group with RAI treatment were much higher than those without RAI after a 40-year follow-up. Next, RAI was associated with an increased risk of SBC in female DTC patients. Especially in the AYA group, the risk increased to 65% when compared with DTC patients without RAI, and the risk increased 21% when compared with the US general population. The risk in the AYA group then increased slightly with latency. After that, patients with RAI had relatively shorter latency of developing SBC compared with patients without RAI. Finally, patients with SBC had poor survival compared with patients with matched only PBC.

A few studies had found an increased risk of SBC in DTC patients, with the SIRs ranging from 1.2 to 2.5, independent of therapy (10–16). Given that malignant breast cancer was thought to be radiogenic, the risks in these studies may be overestimated because most DTC patients would take RAI treatment (5). Previous studies showed conflicting results regarding assessing the risk of SBC after RAI in DTC patients. A study reported an increased SIR of 2.6 after RAI, while the sample size was limited (17). A population-based study from Taiwan demonstrated a slight increase of SBC post-RAI compared with no RAI and controls (18). While most of the studies regarding the association between RAI and SBC in DCT patients showed negative results (5, 12, 13, 19–29). Several reasons could contribute to the variations in results, including patients’ age, different latency selection, not enough follow-up time, statistical methodology, cumulative dose of RAI, and sample size difference.

Age was considered an important risk factor of SPM occurrence after DTC. A population-based study demonstrated that DTC patients aged 20–39 years had the highest SIR of SPM occurrence, and the SIR decreased with increased age (26). Brown et al. (5) found that among thyroid cancer survivors, patients aged 25–49 years had the highest SIR of SBC, and it also decreased with increased age. While in those aged under 25 years, no significant SIR was found. Nevertheless, the factors RAI and age were not considered synchronically in these studies. To the best of our knowledge, this is the first study to analyze the risk of SBC by considering RAI and age synchronically. In this study, in female DTC patients aged 15–39 years, we found a 65% increase of SBC in patients with RAI when compared with patients without RAI, and a 21% increase when compared with the US general population. Whereas in patients aged 40–49 years, only a 18% increase was observed in patients with RAI compared with those without RAI. No increased was found when compared with the US general population. In patients aged ≥70 years, no increased risks were found.

Most of abovementioned studies selected the latency from 2 months to 2 years. Not enough latency after RAI may result in non-RAI related SBC, making unreasonable conclusions in those studies, because at least a 5-year latency from exposure to radiation to the occurrence of SPM was thought to be needed (9, 30, 31). In addition, a few studies selected only 2 months as the minimum latency (5, 22, 24). However, with such a short latency, whether the SBC was synchronous or metachronous could not be clearly differentiated. In this study, at least a 5-year latency after exposure to RAI was guaranteed to better assess RAI-related risk of SBC in female DTC patients. On the other hand, not enough follow-up time may result in contradictory results. Most previous studies had relatively short follow-up time, with a follow-up time of <10 years or between 10 and 20 years (8). Whereas a minimum of 10-year follow-up time is needed to well address the long-term side effects of DTC therapy in children and adolescents (32). In this study, a median follow-up time of 162 months (IQR, 103–259) was reported in the AYA group with RAI, and the maximum follow-up time reached over 40 years. Moreover, we found that the HRs increased slightly with latency in the AYA group, which also suggested that a long-term follow-up should be warranted.

Most of the previous studies assessed the risk by using SIR or relative risk (RR); these methods did not consider the impact of survival on the occurrence of the event of interest. Because part of the patients was newly diagnosed and had not enough follow-up time, the SIR and RR might be inaccurately assessed. In this study, not only SIR but also HR, which was assessed by competing risk regression, were presented. The competing risk analysis considered the impact of competing events, i.e., any reason of death and non-SBC SPMs, on subsequent occurrence of SBC. Hence, the cumulative risk of the event of interest could be estimated in a specific time period, with the considering of the remaining competing events (33, 34).

According to the Radiation Risk Assessment Tool online of the United States National Cancer Institute, a cumulative dose of 2 Gy to the breast in a young female patients could double the lifetime risk for breast cancer (35). In clinic, a few studies assessed the association between RAI dose to the thyroid cancer and the risk of SBC. Rubino et al. found that the SIR of SBC among thyroid cancer patients with ^131^I therapy did not increase, and the RR did not change significantly with the increased of the cumulative dose (21). Ahn et al. demonstrated that, compared with patients with low-dose RAI (<120 mCi), those with high dose (≥120 mCi) had a relative lower risk of subsequent SBC. While no difference was found between patients with and without RAI (25). Though some studies found a trend that the risk of SPM increased with the cumulative dose, no study demonstrated an association between cumulative dose to breast after RAI and the risk of SBC in female DTC patients (26, 27, 29, 36). Based on these studies, it seems that the cumulative dose of RAI is not associated with an increased risk of SBC among female DTC patients. However, these studies also had some shortcomings, such as limited samples, insufficient follow-up, and not considering the effect of age. More studies are warranted to address the issue of the risk of SBC due to exposure to a given dose of RAI among different age populations and provide quantitative information to physicians and patients to make appropriate clinical decision.

Some limitations should be acknowledged in this study. First, surgery treatment is not considered for the risk analysis in this study because, in the SEER database, the information about surgery is blank before year 1998. The lack of adjusting by surgery may cause potential bias in assessing the risk. Second, some possible factors, such as genetic susceptibility, obesity, cumulative dose, and hormones, may also have influence on assessing the risk (8, 11). These factors should be considered in further studies if possible. Third, this is a retrospective study, which lacks randomization and may cause potential biases. Further international, multicenter, observational case-control studies should be performed to better address this issue (37).

## Conclusion

The risk of SBC occurrence increased in female AYA DTC patients treated with RAI compared with those without RAI or US general population, and the risk increased slightly with latency. In addition, the occurrence of SBC worsened the survival. All the findings together provide a meaningful reference and suggest a long-term follow-up of SBC should be performed in female AYA DTC patients treated with RAI.

## Data Availability Statement

The raw data supporting the conclusions of this article will be made available by the authors, without undue reservation.

## Ethics Statement

As this study is a retrospective analysis of public dataset, ethical approval for this study was not required.

## Author Contributions

Conception and design: QY. Administrative support: QY, XZ. Provision of study materials or patients: HZ, MC, and XQ. Collection and assembly of data: MC, GY, YG, and QD. Data analysis and interpretation: XZ, QY, HZ, QD. Manuscript writing: all authors. Final approval of manuscript: all authors. All authors contributed to the article and approved the submitted version.

## Conflict of Interest

The authors declare that the research was conducted in the absence of any commercial or financial relationships that could be construed as a potential conflict of interest.

## Publisher’s Note

All claims expressed in this article are solely those of the authors and do not necessarily represent those of their affiliated organizations, or those of the publisher, the editors and the reviewers. Any product that may be evaluated in this article, or claim that may be made by its manufacturer, is not guaranteed or endorsed by the publisher.

## Supplementary Material

Supplementary Figure 1 A flow chart of the study design. SEER, Surveillance, Epidemiology, and End Results; DTC, differentiated thyroid cancer; ICD-O-3, International Classification of Diseases for Oncology, Third Edition; RAI, radioiodine; SBC, second breast cancer; PSM, propensity score matching; PBC, primary breast cancer.

Supplementary Figure 2 Subgroup analyses by competing risk regression for the risk of developing second breast cancer in adolescent and young adult patients. SHR, subdistribution hazards ratio; CI, confidence interval; SEER, Surveillance, Epidemiology, and End Results.

Supplementary Figure 3 Subgroup analyses by competing risk regression for the risk of developing second breast cancer in middle-aged adult patients. SHR, subdistribution hazards ratio; SEER, Surveillance, Epidemiology, and End Results.

Supplementary Figure 4 Subgroup analyses by competing risk regression for the risk of developing second breast cancer in older adult patients. SHR, subdistribution hazards ratio; SEER, Surveillance, Epidemiology, and End Results.

Supplementary Figure 5 Survival analyses between patients with second breast cancer after no radioiodine and patients with matched only primary breast cancer. **(A)** AYA group, **(B)** middle-aged adult group, and **(C)** older adult group. AYA, adolescent and young adult; RAI, radioiodine; PBC, primary breast cancer; OS, overall survival; NR, not reached; HR, hazard ratio; CI, confidence interval.
