# Supplementary figures and images for: Association of Radioiodine for Differentiated Thyroid Cancer and Second Breast Cancer in Female Adolescent and Young Adult
Source: Front Endocrinol (Lausanne). 2022 Jan 28;12:805194. doi: 10.3389/fendo.2021.805194 (PMC8832493; doi:10.3389/fendo.2021.805194)

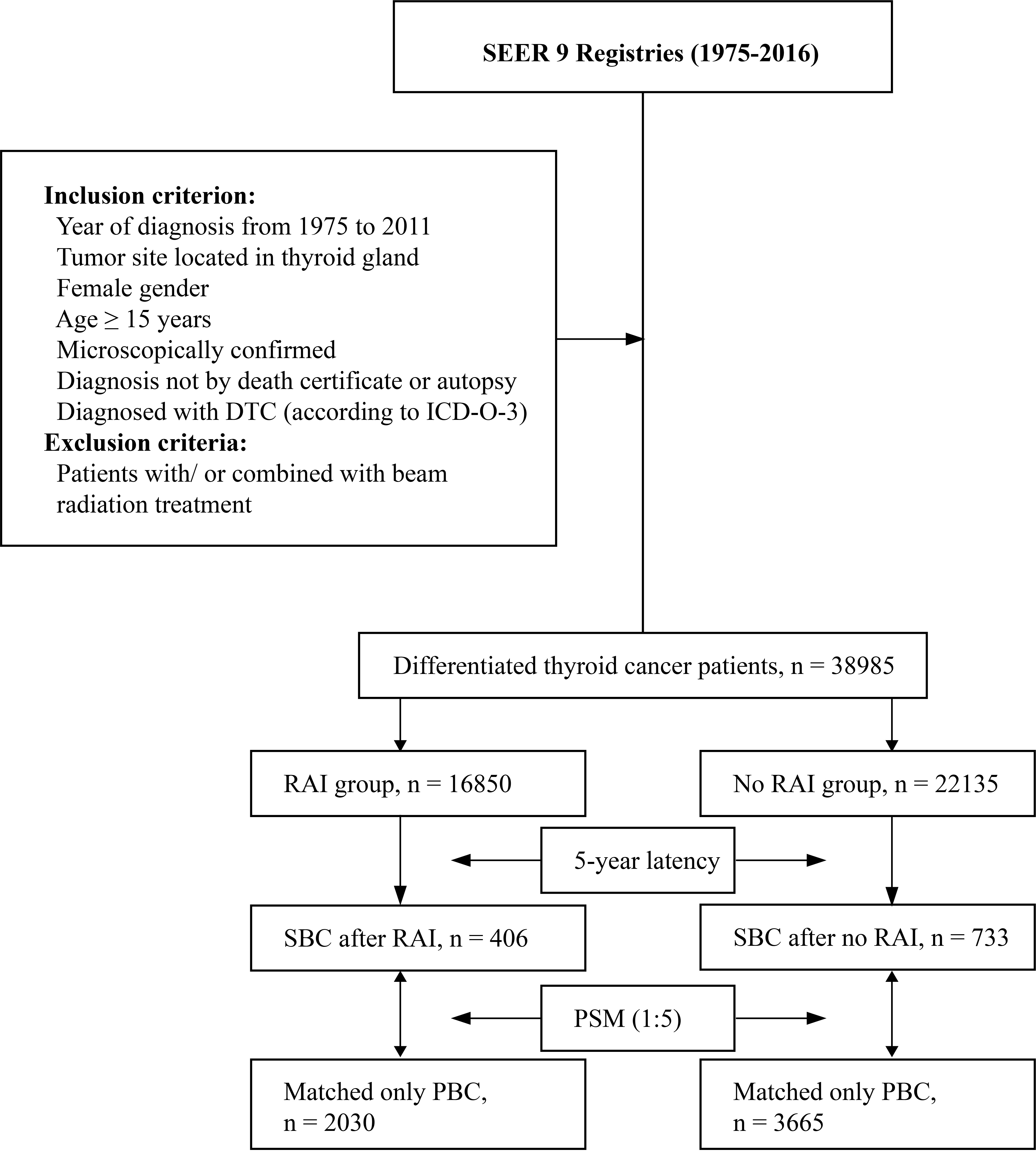

Supplement: Supplementary Figure 1 — A flow chart of the study design. SEER, Surveillance, Epidemiology, and End Results; DTC, differentiated thyroid cancer; ICD-O-3, International Classification of Diseases for Oncology, Third Edition; RAI, radioiodine; SBC, second breast cancer; PSM, propensity score matching; PBC, primary breast cancer. [file Image_1.jpeg]

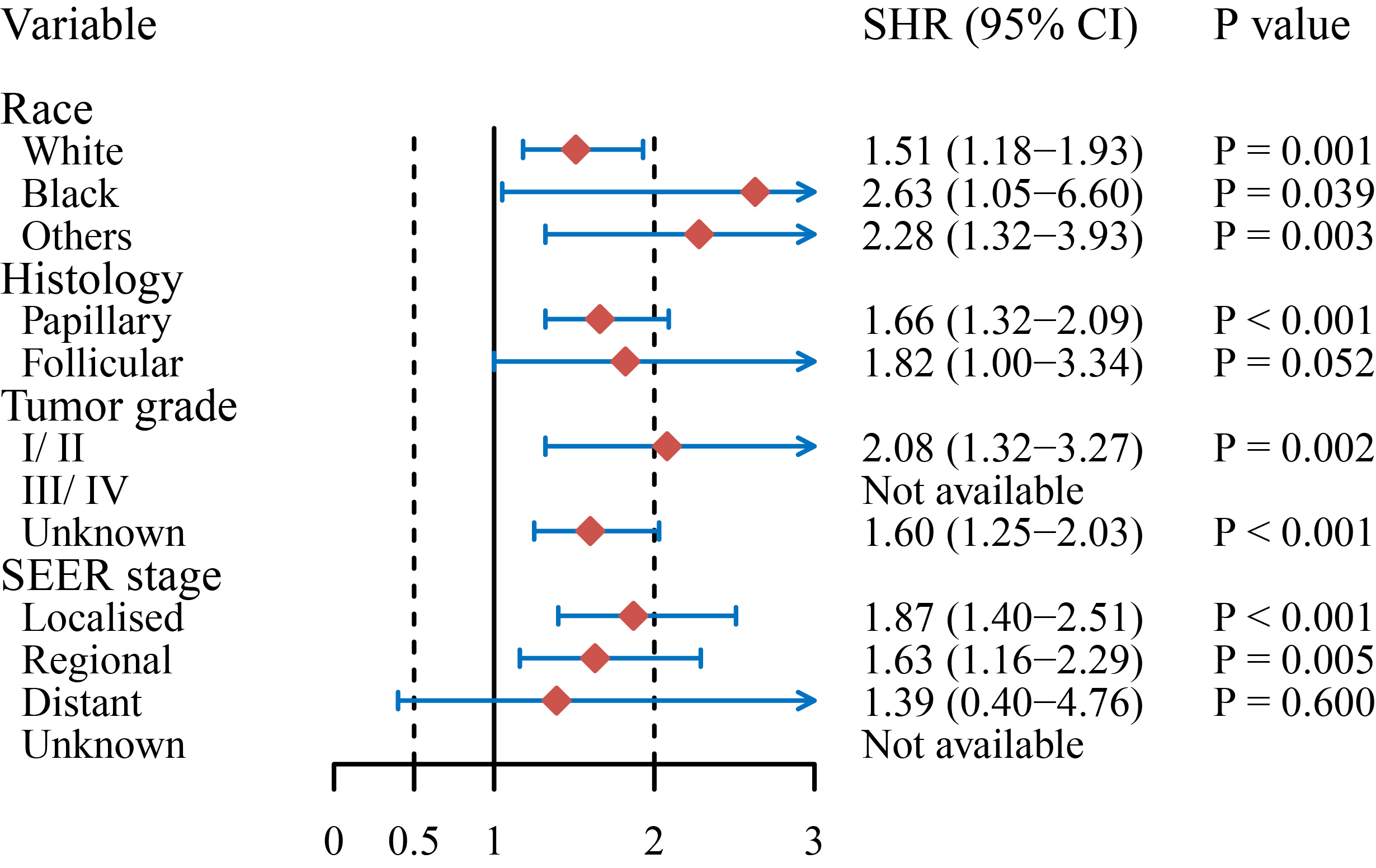

Supplement: Supplementary Figure 2 — Subgroup analyses by competing risk regression for the risk of developing second breast cancer in adolescent and young adult patients. SHR, subdistribution hazards ratio; CI, confidence interval; SEER, Surveillance, Epidemiology, and End Results. [file Image_2.jpeg]

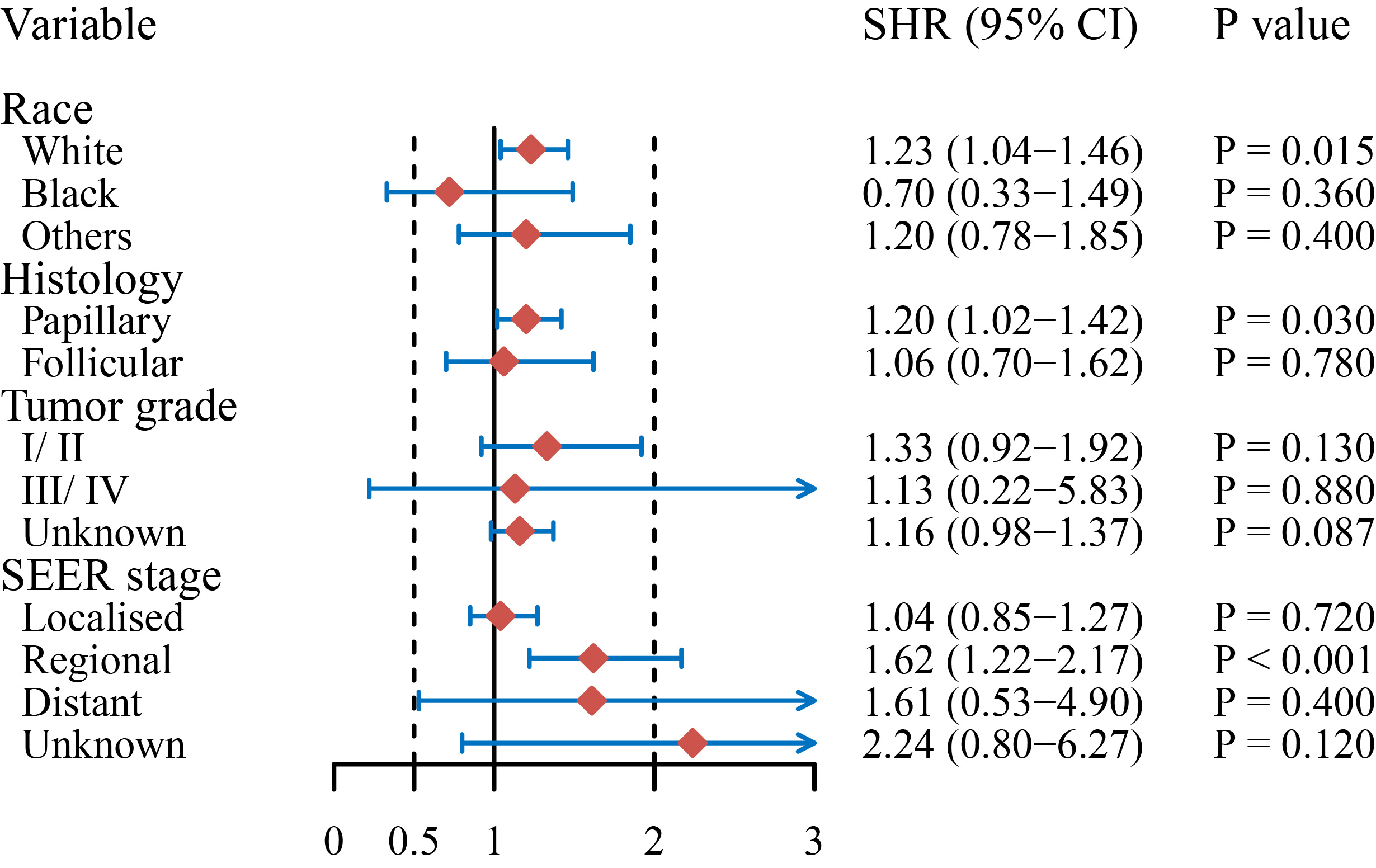

Supplement: Supplementary Figure 3 — Subgroup analyses by competing risk regression for the risk of developing second breast cancer in middle-aged adult patients. SHR, subdistribution hazards ratio; SEER, Surveillance, Epidemiology, and End Results. [file Image_3.jpeg]

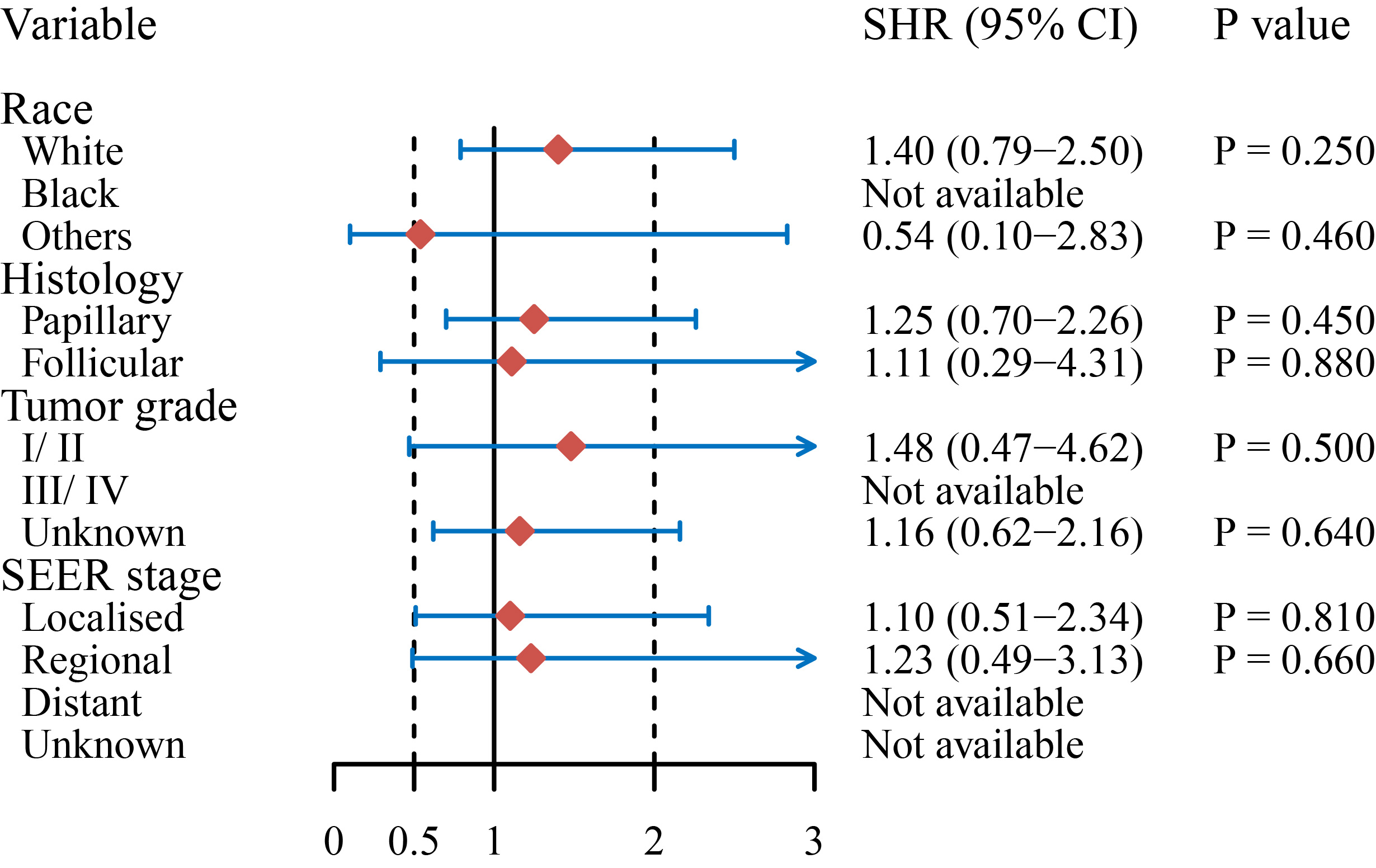

Supplement: Supplementary Figure 4 — Subgroup analyses by competing risk regression for the risk of developing second breast cancer in older adult patients. SHR, subdistribution hazards ratio; SEER, Surveillance, Epidemiology, and End Results. [file Image_4.jpeg]

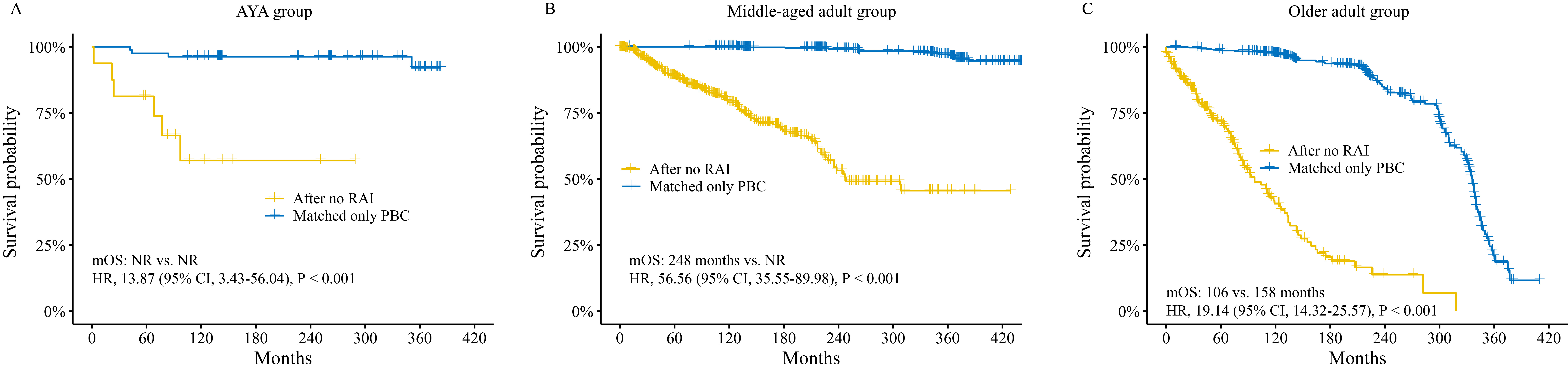

Supplement: Supplementary Figure 5 — Survival analyses between patients with second breast cancer after no radioiodine and patients with matched only primary breast cancer. (A) AYA group, (B) middle-aged adult group, and (C) older adult group. AYA, adolescent and young adult; RAI, radioiodine; PBC, primary breast cancer; OS, overall survival; NR, not reached; HR, hazard ratio; CI, confidence interval. [file Image_5.jpeg]
