# Supplementary material for: Association of Radioiodine for Differentiated Thyroid Cancer and Second Breast Cancer in Female Adolescent and Young Adult
Source: Front Endocrinol (Lausanne). 2022 Jan 28;12:805194. doi: 10.3389/fendo.2021.805194 (PMC8832493; doi:10.3389/fendo.2021.805194)
Supplement: Supplementary file 6 [file Table_1.docx]

Supplementary Table 1: Univariable and multivariable competing risk regression analyses for second breast cancer in all female differentiated thyroid cancer patients.

| **Variable** | **Univariable** | | **Multivariable** | |
| --- | --- | --- | --- | --- |
|  | **HR (95% CI)** | **P value** | **HR (95% CI)** | **P value** |
| **Age (years)** |  |  |  |  |
| 15-39 | Reference | — | Reference | — |
| 40-69 | 2.02 (1.79-2.29) | < 0.001 | 2.02 (1.78-2.28) | < 0.001 |
| ≥ 70 | 1.29 (0.97-1.71) | 0.082 | 1.28 (0.96-1.71) | 0.090 |
| **Race** |  |  |  |  |
| White | Reference | — |  |  |
| Black | 0.99 (0.77-1.28) | 0.960 |  |  |
| Others | 1.06 (0.89-1.27) | 0.520 |  |  |
| **Histology** |  |  |  |  |
| Papillary | Reference | — |  |  |
| Follicular | 0.95 (0.80-1.12) | 0.520 |  |  |
| **Tumor grade** |  |  |  |  |
| I/ II | Reference | — |  |  |
| III/ IV | 0.50 (0.21-1.19) | 0.120 |  |  |
| Unknown | 0.99 (0.85-1.14) | 0.880 |  |  |
| **SEER stage** |  |  |  |  |
| Localised | Reference | — |  |  |
| Regional | 0.91 (0.80-1.03) | 0.140 |  |  |
| Distant | 0.87 (0.59-1.29) | 0.500 |  |  |
| Unknown | 0.88 (0.59-1.32) | 0.540 |  |  |
| **Radioiodine** |  |  |  |  |
| No | Reference | — | Reference | — |
| Yes | 1.28 (1.13-1.44) | < 0.001 | 1.27 (1.13-1.44) | < 0.001 |
| HR, hazard ratio; CI, confidence interval; SEER, Surveillance, Epidemiology, and End Results. | | | | |

Supplementary Table 2: Univariable and multivariable competing risk regression analyses for second breast cancer in female differentiated thyroid cancer patients aged 15-39 years.

| **Variable** | **Univariable** | | **Multivariable** | |
| --- | --- | --- | --- | --- |
|  | **HR (95% CI)** | **P value** | **HR (95% CI)** | **P value** |
| **Race** |  |  |  |  |
| White | Reference | — | Reference | — |
| Black | 1.06 (0.675-1.67) | 0.800 | 1.10 (0.70-1.72) | 0.690 |
| Others | 1.46 (1.092-1.97) | 0.011 | 1.40 (1.05-1.88) | 0.023 |
| **Histology** |  |  |  |  |
| Papillary | Reference | — |  |  |
| Follicular | 1.07 (0.80-1.45) | 0.640 |  |  |
| **Tumor grade** |  |  |  |  |
| I/ II | Reference | — |  |  |
| III/ IV | 0.28 (0.04-2.05) | 0.210 |  |  |
| Unknown | 0.94 (0.73-1.19) | 0.590 |  |  |
| **SEER stage** |  |  |  |  |
| Localised | Reference | — |  |  |
| Regional | 1.08 (0.87-1.33) | 0.500 |  |  |
| Distant | 0.85 (0.44-1.65) | 0.630 |  |  |
| Unknown | 0.76 (0.35-1.62) | 0.470 |  |  |
| **Radioiodine** |  |  |  |  |
| No | Reference | — | Reference | — |
| Yes | 1.68 (1.35-2.08) | < 0.001 | 1.65 (1.33-2.05) | < 0.001 |
| HR, hazard ratio; CI, confidence interval; SEER, Surveillance, Epidemiology, and End Results. | | | | |

Supplementary Table 3: Univariable competing risk regression analysis for second breast cancer in female differentiated thyroid cancer patients aged 40-69 years.

| **Variable** | **Univariable** | |  |
| --- | --- | --- | --- |
|  | **HR (95% CI)** | **P value** |  |
| **Race** |  |  |  |
| White | Reference | — |  |
| Black | 0.98 (0.71-1.34) | 0.880 |  |
| Others | 0.86 (0.68-1.09) | 0.210 |  |
| **Histology** |  |  |  |
| Papillary | Reference | — |  |
| Follicular | 0.86 (0.69-1.06) | 0.160 |  |
| **Tumor grade** |  |  |  |
| I/ II | Reference | — |  |
| III/ IV | 0.75 (0.28-1.96) | 0.550 |  |
| Unknown | 1.01 (0.83-1.22) | 0.950 |  |
| **SEER stage** |  |  |  |
| Localised | Reference | — |  |
| Regional | 0.91 (0.77-1.07) | 0.260 |  |
| Distant | 1.06 (0.63-1.78) | 0.830 |  |
| Unknown | 0.98 (0.59-1.63) | 0.950 |  |
| **Radioiodine** |  |  |  |
| No | Reference | — |  |
| Yes | 1.18 (1.01-1.38) | 0.032 |  |
| HR, hazard ratio; CI, confidence interval; SEER, Surveillance, Epidemiology, and End Results. | | | |

Supplementary Table 4: Univariable competing risk regression analysis for second breast cancer in female differentiated thyroid cancer patients aged ≥ 70 years.

| **Variable** | **Univariable** | |  |
| --- | --- | --- | --- |
|  | **HR (95% CI)** | **P value** |  |
| **Race** |  |  |  |
| White | Reference | — |  |
| Black | 0.31 (0.04-2.27) | 0.250 |  |
| Others | 0.89 (0.38-2.08) | 0.790 |  |
| **Histology** |  |  |  |
| Papillary | Reference | — |  |
| Follicular | 0.68 (0.33-1.40) | 0.300 |  |
| **Tumor grade** |  |  |  |
| I/ II | — | — |  |
| III/ IV | — | — |  |
| Unknown | — | — |  |
| **SEER stage** |  |  |  |
| Localised | Reference | — |  |
| Regional | 0.93 (0.52-1.66) | 0.800 |  |
| Distant | 1.11 (0.27-4.62) | 0.890 |  |
| Unknown | 1.38 (0.33-5.75) | 0.650 |  |
| **Radioiodine** |  |  |  |
| No | Reference | — |  |
| Yes | 1.24 (0.72-2.13) | 0.440 |  |
| HR, hazard ratio; CI, confidence interval; SEER, Surveillance, Epidemiology, and End Results. | | | |

Supplementary Table 5: Standardized incidence ratios of second breast cancer after radioiodine in female differentiated thyroid cancer patients compared to US general population.

| Age group | Latency | Observed | Expected | SIR | 95% CI | |
| --- | --- | --- | --- | --- | --- | --- |
|  |  |  |  |  | Lower | Upper |
| Adolescent and young adult | 60-119 months | 35 | 25.96 | 1.35 | 0.94 | 1.88 |
|  | 120-179 months | 31 | 27.14 | 1.14 | 0.78 | 1.62 |
|  | 180-239 months | 25 | 23.48 | 1.06 | 0.69 | 1.57 |
|  | 240-299 months | 18 | 17.38 | 1.04 | 0.61 | 1.64 |
|  | 300-359 months | 14 | 10.87 | 1.29 | 0.70 | 2.16 |
|  | ≥ 360 months | 13 | 7.14 | 1.82 | 0.97 | 3.12 |
| Middle-aged adult | 60-119 months | 117 | 103.86 | 1.13 | 0.93 | 1.35 |
|  | 120-179 months | 67 | 61.90 | 1.08 | 0.84 | 1.37 |
|  | 180-239 months | 34 | 34.70 | 0.98 | 0.68 | 1.37 |
|  | 240-299 months | 19 | 17.62 | 1.08 | 0.65 | 1.68 |
|  | 300-359 months | 7 | 7.36 | 0.95 | 0.38 | 1.96 |
|  | ≥ 360 months | 5 | 2.98 | 1.68 | 0.54 | 3.92 |
| Older adult | 60-119 months | 17 | 11.20 | 1.52 | 0.88 | 2.43 |
|  | 120-179 months | 2 | 4.09 | 0.49 | 0.05 | 1.77 |
|  | 180-239 months | 2 | 1.12 | 1.79 | 0.20 | 6.47 |
|  | 240-299 months | 0 | 0.28 | 0 | 0 | 13.22 |
|  | 300-359 months | 0 | 0.05 | 0 | 0 | 72.96 |
|  | ≥ 360 months | 0 | 0 | 0 | 0 | 1207.81 |
| SIR, standardized incidence ratio; CI, confidence interval. | | | | | | |

Supplementary Table 6: Standardized incidence ratio s of second breast cancer after no radioiodine in female differentiated thyroid cancer patients compared to US general population.

| Age group | Latency | Observed | Expected | SIR | 95% CI | |
| --- | --- | --- | --- | --- | --- | --- |
|  |  |  |  |  | Lower | Upper |
| Adolescent and young adult | 60-119 months | 45 | 34.36 | 1.31 | 0.96 | 1.75 |
|  | 120-179 months | 41 | 43.45 | 0.97 | 0.69 | 1.31 |
|  | 180-239 months | 49 | 44.92 | 1.09 | 0.81 | 1.44 |
|  | 240-299 months | 36 | 43.06 | 0.84 | 0.59 | 1.16 |
|  | 300-359 months | 32 | 36.95 | 0.87 | 0.59 | 1.22 |
|  | ≥ 360 months | 38 | 41.57 | 0.91 | 0.65 | 1.25 |
| Middle-aged adult | 60-119 months | 166 | 149.03 | 1.11 | 0.95 | 1.30 |
|  | 120-179 months | 122 | 109.07 | 1.12 | 0.93 | 1.34 |
|  | 180-239 months | 73 | 76.72 | 0.95 | 0.75 | 1.20 |
|  | 240-299 months | 54 | 51.23 | 1.05 | 0.79 | 1.38 |
|  | 300-359 months | 30 | 30.04 | 1.00 | 0.67 | 1.43 |
|  | ≥ 360 months | 13 | 18.81 | 0.69 | 0.37 | 1.18 |
| Older adult | 60-119 months | 23 | 21.38 | 1.08 | 0.68 | 1.61 |
|  | 120-179 months | 8 | 9.20 | 0.87 | 0.37 | 1.71 |
|  | 180-239 months | 3 | 3.22 | 0.93 | 0.19 | 2.72 |
|  | 240-299 months | 0 | 0.98 | 0 | 0 | 3.76 |
|  | 300-359 months | 0 | 0.18 | 0 | 0 | 20.93 |
|  | ≥ 360 months | 0 | 0.03 | 0 | 0 | 125.08 |
| SIR, standardized incidence ratio; CI, confidence interval. | | | | | | |

Supplementary Table 7: Clinical features comparison between second breast cancer patients after radioiodine and matched only primary breast cancer patients.

| **Variables** | **Total,**  **N=2436 (%)** | **Matched only PBC, N=2030 (%)** | **SBC after RAI, N=406 (%)** | **P value** |
| --- | --- | --- | --- | --- |
| **Age at breast cancer diagnosis (years)** |  |  |  | 1.000 |
| 15-39 | 96 (4) | 80 (4) | 16 (4) |  |
| 40-69 | 1770 (73) | 1475 (73) | 295 (73) |  |
| ≥ 70 | 570 (23) | 475 (23) | 95 (23) |  |
| **Race** |  |  |  | 1.000 |
| White | 1932 (79) | 1610 (79) | 322 (79) |  |
| Black | 102 (4) | 85 (4) | 17 (4) |  |
| Others | 402 (17) | 335 (17) | 67 (17) |  |
| **Tumor grade** |  |  |  | 1.000 |
| I | 468 (19) | 390 (19) | 78 (19) |  |
| II | 1146 (47) | 955 (47) | 191 (47) |  |
| III | 594 (24) | 495 (24) | 99 (24) |  |
| IV | 24 (1) | 20 (1) | 4 (1) |  |
| Unknown | 204 (8) | 170 (8) | 34 (8) |  |
| **SEER stage** |  |  |  | 1.000 |
| Localised | 1374 (56) | 1145 (56) | 229 (56) |  |
| Regional | 672 (28) | 560 (28) | 112 (28) |  |
| Distant | 66 (3) | 55 (3) | 11 (3) |  |
| Unknown | 324 (13) | 270 (13) | 54 (13) |  |
| PBC, primary breast cancer; SBC, second breast cancer; RAI, radioiodine; SEER, Surveillance, Epidemiology, and End Results. | | | | |

Supplementary Table 8: Clinical features comparison between second breast cancer patients after no radioiodine and matched only primary breast cancer patients.

| **Variables** | **Total,**  **N=4398 (%)** | **Matched only PBC, N=3665 (%)** | **SBC after no RAI, N=733 (%)** | **P value** |
| --- | --- | --- | --- | --- |
| **Age at breast cancer diagnosis (years)** |  |  |  | 1.000 |
| 15-39 | 96 (2) | 80 (2) | 16 (2) |  |
| 40-69 | 3030 (69) | 2525 (69) | 505 (69) |  |
| ≥ 70 | 1272 (29) | 1060 (29) | 212 (29) |  |
| **Race** |  |  |  | 1.000 |
| White | 3702 (84) | 3085 (84) | 617 (84) |  |
| Black | 270 (6) | 225 (6) | 45 (6) |  |
| Others | 426 (10) | 355 (10) | 71 (10) |  |
| **Tumor grade** |  |  |  | 1.000 |
| I | 822 (19) | 685 (19) | 137 (19) |  |
| II | 1752 (40) | 1460 (40) | 292 (40) |  |
| III | 1176 (27) | 980 (27) | 196 (27) |  |
| IV | 72 (2) | 60 (2) | 12 (2) |  |
| Unknown | 576 (13) | 480 (13) | 96 (13) |  |
| **SEER stage** |  |  |  | 1.000 |
| Localised | 2592 (59) | 2160 (59) | 432 (59) |  |
| Regional | 1332 (30) | 1110 (30) | 222 (30) |  |
| Distant | 150 (3) | 125 (3) | 25 (3) |  |
| Unknown | 324 (7) | 270 (7) | 54 (7) |  |
| PBC, primary breast cancer; SBC, second breast cancer; RAI, radioiodine; SEER, Surveillance, Epidemiology, and End Results. | | | | |
